# High temperature promotes amyloid β-protein production and γ-secretase complex formation via Hsp90

**DOI:** 10.1074/jbc.RA120.013845

**Published:** 2021-01-13

**Authors:** Arshad Ali Noorani, Hitoshi Yamashita, Yuan Gao, Sadequl Islam, Yang Sun, Tomohisa Nakamura, Hiroyuki Enomoto, Kun Zou, Makoto Michikawa

**Affiliations:** 1 Department of Biochemistry, Graduate School of Medical Sciences, Nagoya City University, Nagoya, Japan; 2 Department of Biomedical Sciences, College of Life and Health Sciences, Chubu University, Kasugai, Japan

**Keywords:** amyloid β-protein, Alzheimer's disease, temperature, heat shock protein 90 (Hsp90), γ-secretase, Alzheimer disease, amyloid-beta (AB), gamma-secretase, presenilin

## Abstract

Alzheimer's disease (AD) is characterized by neuronal loss and accumulation of β-amyloid-protein (Aβ) in the brain parenchyma. Sleep impairment is associated with AD and affects about 25–40% of patients in the mild-to-moderate stages of the disease. Sleep deprivation leads to increased Aβ production; however, its mechanism remains largely unknown. We hypothesized that the increase in core body temperature induced by sleep deprivation may promote Aβ production. Here, we report temperature-dependent regulation of Aβ production. We found that an increase in temperature, from 37 °C to 39 °C, significantly increased Aβ production in amyloid precursor protein-overexpressing cells. We also found that high temperature (39 °C) significantly increased the expression levels of heat shock protein 90 (Hsp90) and the C-terminal fragment of presenilin 1 (PS1-CTF) and promoted γ-secretase complex formation. Interestingly, Hsp90 was associated with the components of the premature γ-secretase complex, anterior pharynx-defective-1 (APH-1), and nicastrin (NCT) but was not associated with PS1-CTF or presenilin enhancer-2. Hsp90 knockdown abolished the increased level of Aβ production and the increased formation of the γ-secretase complex at high temperature in culture. Furthermore, with *in vivo* experiments, we observed increases in the levels of Hsp90, PS1-CTF, NCT, and the γ-secretase complex in the cortex of mice housed at higher room temperature (30 °C) compared with those housed at standard room temperature (23 °C). Our results suggest that high temperature regulates Aβ production by modulating γ-secretase complex formation through the binding of Hsp90 to NCT/APH-1.

Alzheimer's disease (AD) is a progressive and multifactorial neurodegenerative disorder of the central nervous system (1). Two major features characterize the AD patient's brain. One is the accumulation of amyloid plaques between nerve cells, made up of β-amyloid-protein (Aβ) fibrils surrounded by degenerating neurons (2). The second is neurofibrillary tangles, which are composed of hyperphosphorylated tau protein aggregates inside neurons (3). AD is thought to be caused by an imbalance between Aβ production and clearance, which leads to increased formation of Aβ oligomers, insoluble fibrils, and plaques in the brain parenchyma that initially damage synapses and later cause neurodegeneration (2).

Aβ peptides are 37–43 amino acids in length and are derived from proteolytic cleavage of the transmembrane protein amyloid precursor protein (APP) by β-secretase, also called β-site APP-cleaving enzyme 1 (BACE1), and an aspartyl protease, γ-secretase (4). γ-Secretase is a high-molecular weight complex that minimally consists of four core subunits: presenilin (PS1 and PS2), nicastrin (NCT), anterior pharynx-defective-1 (APH-1), and presenilin enhancer-2 (PEN-2) (5). PS is the catalytic subunit of the enzyme complex, whereas NCT functions in substrate recognition. APH-1 functions as a stabilizing scaffold in the assembly of the complex, forming a subcomplex with NCT, and PEN-2 plays a central role in PS endoproteolysis, which generates cleaved PS N-terminal fragment and PS C-terminal fragment (6). However, despite its important role in Aβ production, the regulatory mechanism underlying the formation and activity of the γ-secretase complex is largely unknown.

The majority of AD cases are likely due to lifestyle and environmental factors that affect the brain over time. Patients with AD exhibit significant noncognitive behavioral symptoms, such as depression, hallucination, agitation, weight loss, hyperactivity, sleep–wake cycle disturbances, and neuroendocrine alterations attributable to hypothalamic dysfunction (7). Noncognitive changes in AD patients include disruptions of the normal circadian rest-activity rhythm and body temperature rhythm, usually followed by an increase in nocturnal activity and a raised body temperature (8, 9, 10, 11, 12, 13). These lines of evidence suggest a potential role for body temperature in the development of AD. A close relationship between sleep and body temperature has been seen frequently in humans. In the normal human circadian cycle, sleep occurs when the core body temperature decreases (14, 15). In experimental mice, the body temperature drops during the light (resting) phase and rises during the dark (active) phase. Sleep deprivation worsens Aβ pathology in transgenic mouse models, and administration of an orexin antagonist to increase sleep reduces amyloid plaque burden (16). These studies indicate that Aβ levels are closely associated with sleep, but the exact mechanisms involved in this process have still not been identified. A recent study on humans suggests that one night of sleep deprivation leads to an increase in Aβ production of about 5% (17).

We hypothesized that sleep and circadian disturbances lead to increased core body temperature, which results in enhancement of Aβ production. Here, we examined Aβ production in human embryonic kidney cells that overexpress human APP (HEK-APP cells) and found that high temperature increased Aβ production. In addition, γ-secretase complex formation and activity were significantly increased in the brains of mice housed at 30 °C compared with 23 °C. In this study, we showed for the first time that Hsp90 interacted with NCT and APH-1 at high temperature and promoted γ-secretase complex formation and activity, leading to enhanced Aβ production.

## Results

### High temperature increases Aβ production and modulates γ-secretase activity

**Figure 1:**
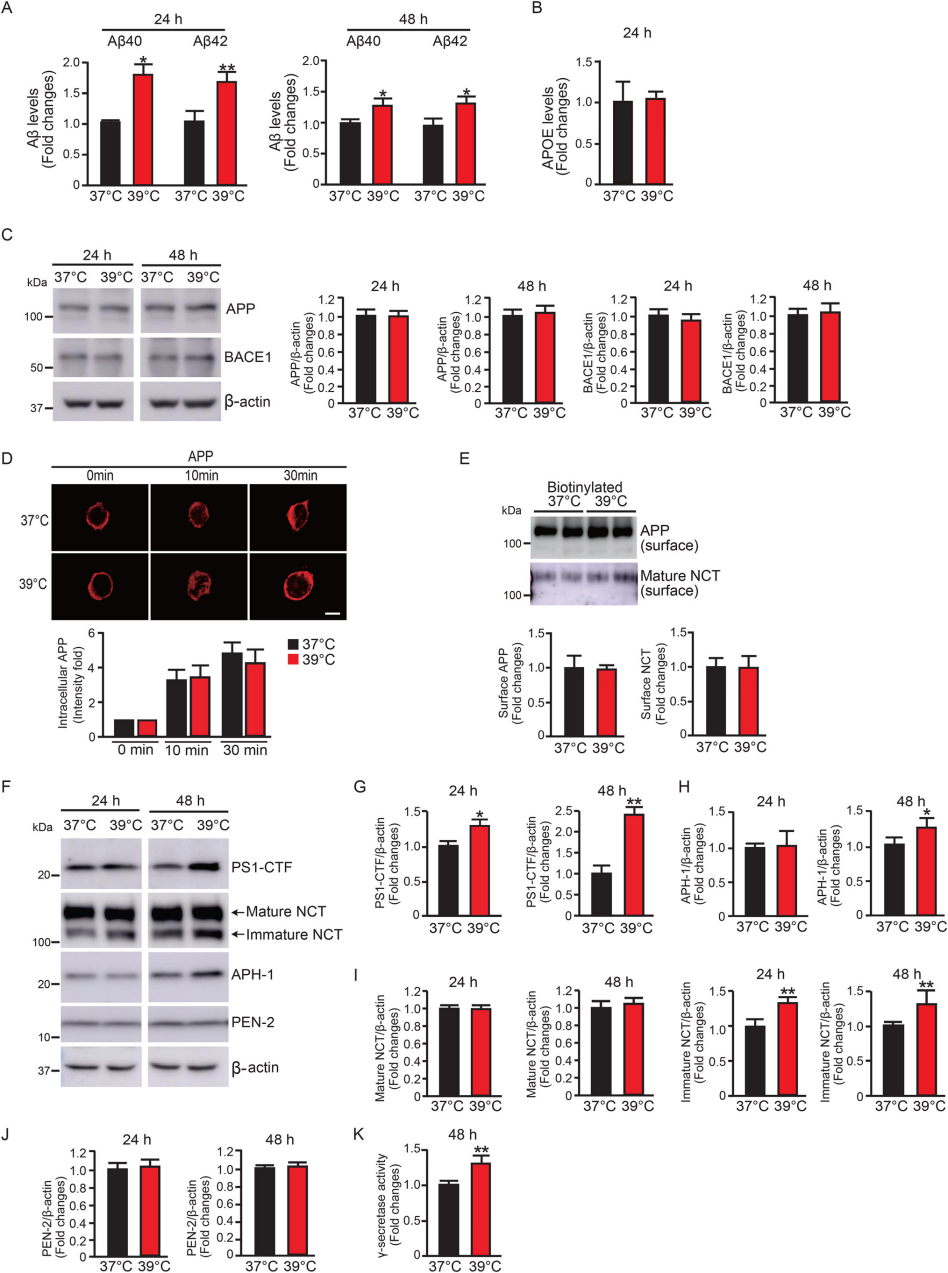
**High temperature increases Aβ production and the levels of** γ**-secretase components.***A*, high temperature increased Aβ production in HEK-APP cells. The levels of Aβ40 and Aβ42 in HEK-APP cells were measured with an Aβ ELISA kit. *B*, ApoE levels in HEK-APP cells were measured with an ApoE ELISA kit. *C*, the expression levels of full-length APP and BACE1 in HEK-APP cells at 24 and 48 h were detected with Western blotting. *D*, APP immunostaining showed similar internalization of APP at 37 or 39 °C. *E*, biotinylated surface APP and NCT in HEK-APP cells at 37 or 39 °C were detected with Western blotting. *F–J*, PS1-CTF, NCT, APH-1, and PEN-2 levels in HEK-APP cells at 24 and 48 h were detected with Western blotting. HEK-APP cells cultured at 39 °C showed significantly increased levels of PS1-CTF (*G*) and immature NCT (*I*) at 24 and 48 h and increased levels of APH-1 (*H*) at 48 h compared with cells cultured at 37 °C. *K*, high temperature increased γ-secretase activity detected with a fluorogenic substrate. Data are the mean ± S.E. (*error bars*) from three independent experiments. *, *p* < 0.05; **, *p* < 0.01 as determined with Student's *t* test. *Scale bar* (*D*), 5 μm.

To study whether high temperature plays a role in Aβ production, we compared Aβ production in HEK-APP cells cultured at 37 and 39 °C. We found that an increase in temperature, from 37 to 39 °C, enhanced the production of Aβ40 1.7-fold and Aβ42 1.6-fold after 24 h in culture. The increase in Aβ40 and Aβ42 production was also observed after 48 h in HEK-APP cells cultured at 39 °C compared with that of cells cultured at 37 °C (Fig. 1*A*). These results indicate that temperature can regulate Aβ production and that high temperature promotes Aβ production. In contrast, the levels of another secretory protein, ApoE, did not change after 24 h in culture at 39 °C compared with those at 37 °C (Fig. 1*B*), suggesting that a higher temperature did not change the rate of protein secretion. Because Aβ is generated from APP by β-secretase (BACE1) and γ-secretase, we then examined the expression levels of APP, BACE1, and the four components of the γ-secretase complex in HEK-APP cells cultured at 39 °C. The internalization rate and cell surface expression of APP were also examined.

The expression levels of full-length APP and BACE1 showed no difference between cells cultured at 37 and 39 °C for 24 or 48 h, suggesting that APP and BACE1 are not involved in the increase in Aβ production by high temperature (Fig. 1*C*). The internalization of APP did not change in the cells cultured at 39 °C compared with the cells cultured at 37 °C (Fig. 1*D*), and the cell surface expression levels of APP and NCT were also not altered by a higher temperature, 39 °C (Fig. 1*E*). These results suggest that APP transport or internalization of the substrate was not altered by a higher temperature. We further investigated the expression levels of the four components of the γ-secretase complex, namely PS1, NCT, APH-1, and PEN-2. We found that the expression levels of PS1-CTF increased 1.3- and 2.3-fold in the cells cultured at 39 °C for 24 and 48 h, respectively, compared with the cells cultured at 37 °C (Fig. 1, *F* and *G*). The level of APH-1 was slightly increased in the cells cultured at 39 °C for 48 h (Fig. 1, *F* and *H*). The expression levels of mature NCT were not affected, whereas immature NCT levels increased 1.2- and 1.3-fold in the cells cultured at 39 °C for 24 and 48 h, respectively (Fig. 1, *F* and *I*). PEN-2 expression levels were not affected by the high temperature (Fig. 1, *F* and *J*). We also found that γ-secretase activity increased in the cells cultured at 39 °C for 48 h by an *in vitro* γ-secretase activity assay (Fig. 1*K*). These results suggest that high temperature increases the expression levels of components of γ-secretase, including PS1-CTF, APH-1, and immature NCT, and enhances γ-secretase activity and Aβ production.

**Figure 2:**
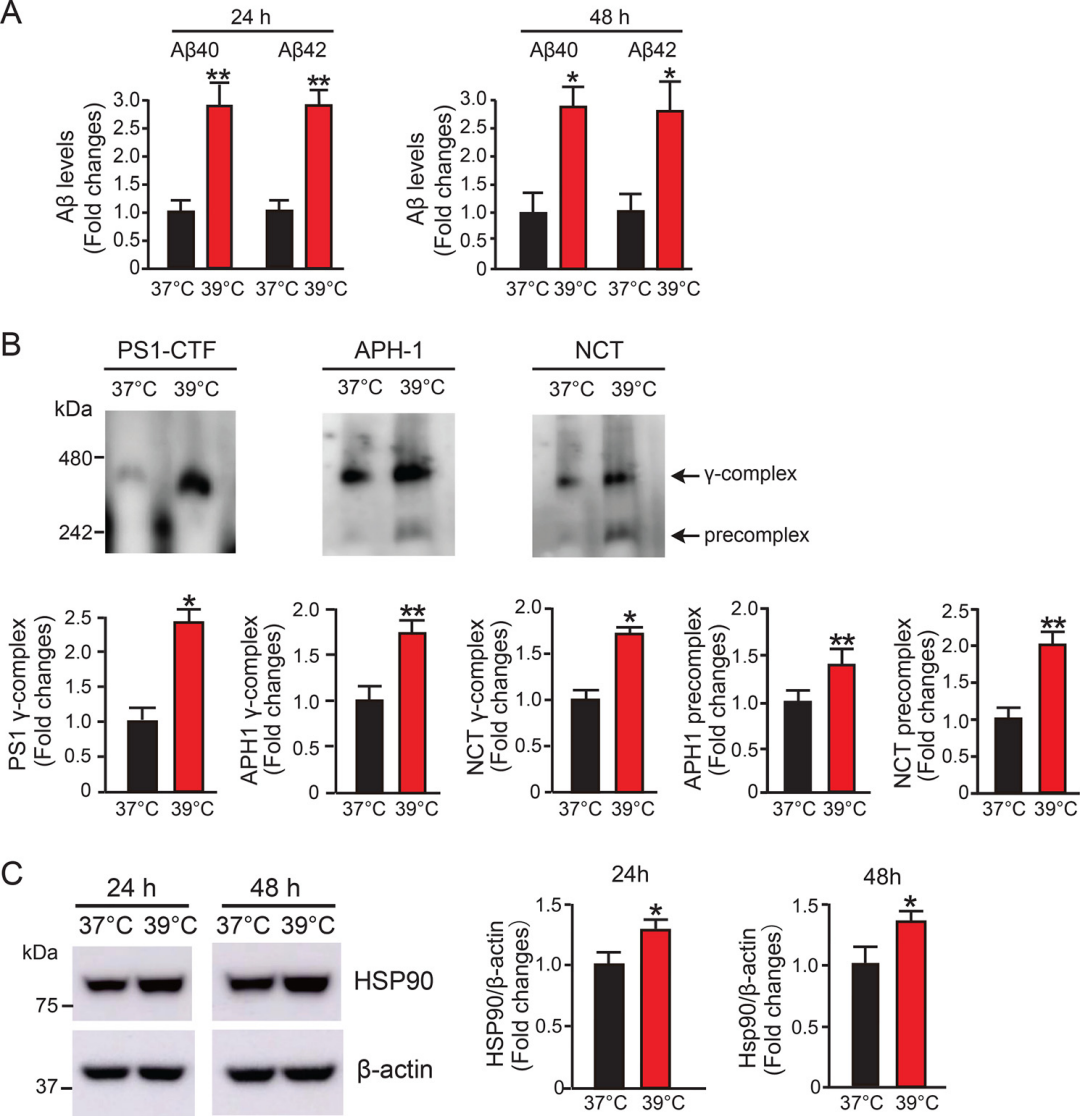
**High temperature promotes** γ**-secretase activity and complex formation and enhances Hsp90 expression.***A*, the levels of Aβ40 and Aβ42 in the culture media of HEK-C99 cells were measured with an Aβ ELISA kit. High temperature (39 °C) increased Aβ40 and Aβ42 production in HEK-C99 cells at 24 and 48 h. *B*, 5 μg of protein from HEK-APP cell lysates was subjected to BN-PAGE and analyzed with immunoblotting using PS1-CTF (*left*), APH-1 (*middle*), and NCT antibodies (*right*). High temperature significantly increased levels of the mature complex as well as the precomplex of γ-secretase at 24 h. *C*, the expression level of Hsp90 in HEK-APP cells was detected with Western blotting. The Hsp90 level was significantly increased at 39 °C. Data are the mean ± S.E. (*error bars*) from three independent experiments. *, *p* < 0.05; **, *p* < 0.01 as determined with Student's *t* test.

To confirm this, we investigated Aβ production using HEK cells overexpressing C99, which is the C-terminal fragment of APP after β-cleavage by BACE1 and is a substrate for γ-secretase. As expected, we also found that Aβ40 and Aβ42 production was increased more than 2-fold in HEK-C99 cells cultured at 39 °C compared with that of cells cultured at 37 °C for 24 or 48 h (Fig. 2*A*). These results indicate that the increased levels of Aβ at 39 °C resulted from enhanced γ-secretase activity.

### High temperature promotes γ-secretase complex formation and enhances Hsp90 expression

We next examined the potential effect of high temperature on γ-secretase complex assembly. We solubilized HEK-APP cells in digitonin, because this detergent not only preserves the γ-secretase complex but is compatible with γ-secretase activity (18). The mature γ-secretase complex band is ∼440 kDa, and the APH-1/NCT precomplex band is ∼250 kDa (19). NCT and APH-1 form a stable precomplex prior to the formation of the mature γ-secretase complex (20). We found that the level of γ-secretase complex detected with the PS1-CTF antibody at ∼440 kDa was significantly increased 2.5-fold in the cells cultured at 39 °C for 24 h compared with at 37 °C (Fig. 2*B*, *left*). The precomplex level of γ-secretase, showing formation of the intermediate APH-1/NCT precomplex at ∼250 kDa, also increased 1.4-fold and 2-fold, respectively, at 39 °C compared with 37 °C, as indicated by blotting with APH-1 and NCT antibodies (Fig. 2*B*, *middle* and *right panels*). The γ-secretase complex at ∼440 kDa detected by APH-1 and NCT antibodies also showed significant increases in the cells cultured at 39 °C (Fig. 2*B*, *middle* and *right panels*). Because Hsp90 is a key molecule in response to temperature change, we examined whether Hsp90 is up-regulated and involved in the formation of γ-secretase complex and enhanced Aβ production. We found that the expression levels of Hsp90 were markedly increased in HEK-APP cells cultured at 39 °C for 24 and 48 h compared with 37 °C (Fig. 2*C*).

### Interaction of Hsp90 with NCT and APH-1

**Figure 3:**
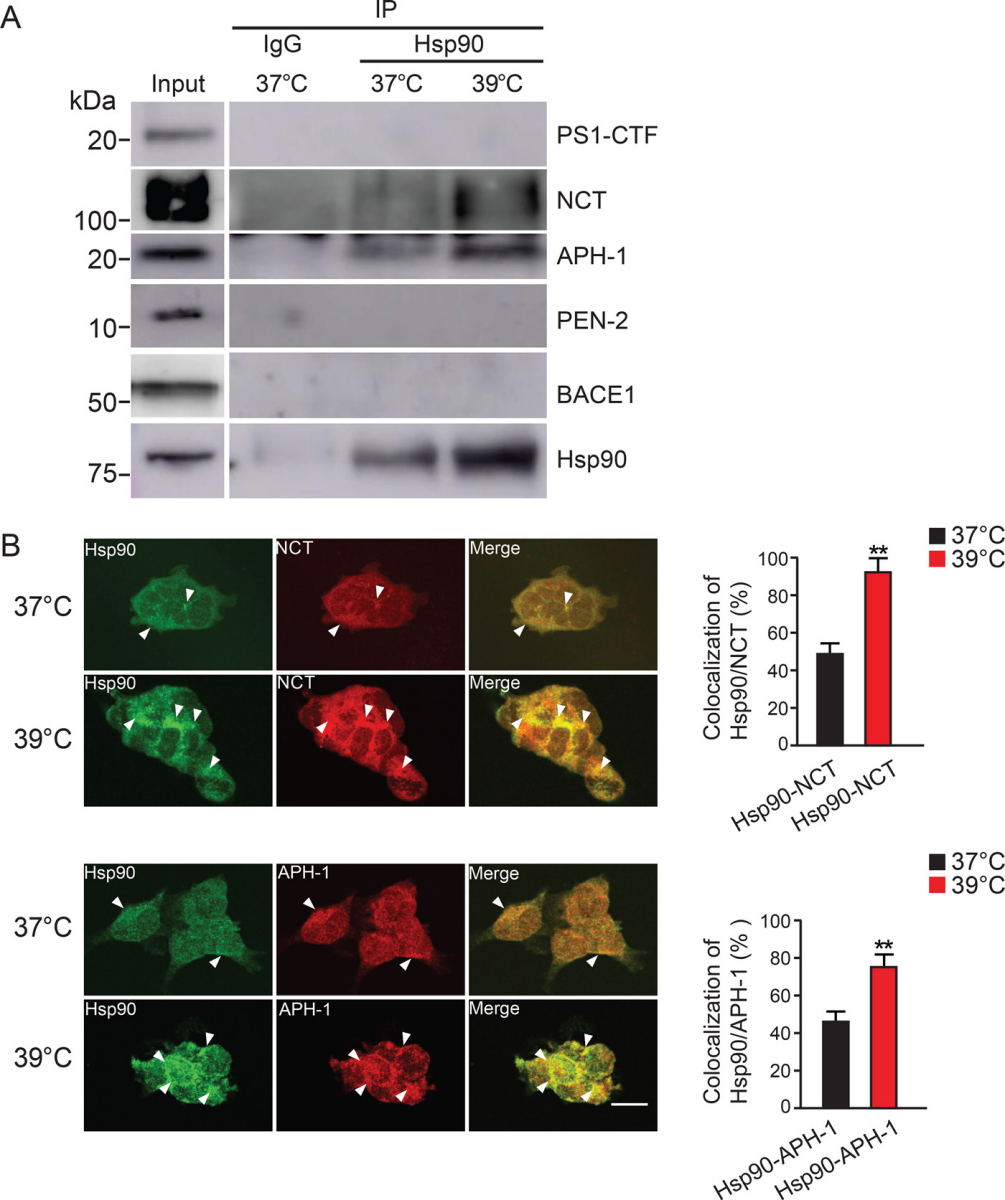
**Hsp90 interacts with NCT and APH-1, and these interactions are promoted at high temperature.***A*, lysates from HEK-APP cells at 48 h were immunoprecipitated with the Hsp90 antibody. The obtained samples were subjected to Western blotting and analyzed using antibodies that recognize γ-secretase components. Hsp90 interacted with NCT and APH-1 but not with PS1-CTF or PEN-2 at 37 or 39 °C. *B*, immunofluorescence analysis showed that Hsp90 colocalized with APH-1 or NCT (*arrowheads*). HEK-APP cells were stained with secondary antibodies labeled with Alexa Fluor 488 and Alexa Fluor 568: Hsp90 (*green*), APH-1 (*red*), and NCT (*red*). *Scale bar*, 20 μm. Data are the mean ± S.E. (*error bars*) from three independent experiments. **, *p* < 0.01 as determined with Student's *t* test.

To examine whether Hsp90 is involved in the formation of γ-secretase complex, we performed immunoprecipitation (IP) experiments. We found that Hsp90 bound to NCT and APH-1, but not the other γ-secretase components, PS1-CTF or PEN-2. In addition, culturing at 39 °C increased the binding of Hsp90 to NCT and APH-1 compared with that of cells cultured at 37 °C (Fig. 3*A*). These results suggest that Hsp90 is involved in formation of the γ-secretase complex. Confocal microscopy studies demonstrated that the expression level of Hsp90 was increased in the high-temperature condition (39 °C) compared with 37 °C (Fig. 3*B*, *left panels*) and that the colocalization of Hsp90 with NCT/APH-1 was significantly enhanced (Fig. 3*B*, *right panels*, *merge*). The percentage of Hsp90 colocalization with NCT and APH-1 increased to 1.8- and 1.6-fold, respectively, at 39 °C compared with those at 37 °C (Fig. 3*B*, *bar graphs*). These results are consistent with the findings in the IP studies. At 37 °C, we also detected a lower degree of colocalization of Hsp90 with NCT or APH-1. Taken together, these results suggest that Hsp90 at high temperature may promote γ-secretase complex assembly, possibly first by formation of the precomplex, through its interaction with NCT and APH-1.

### Knockdown of Hsp90 reduces Aβ production at high temperature

**Figure 4:**
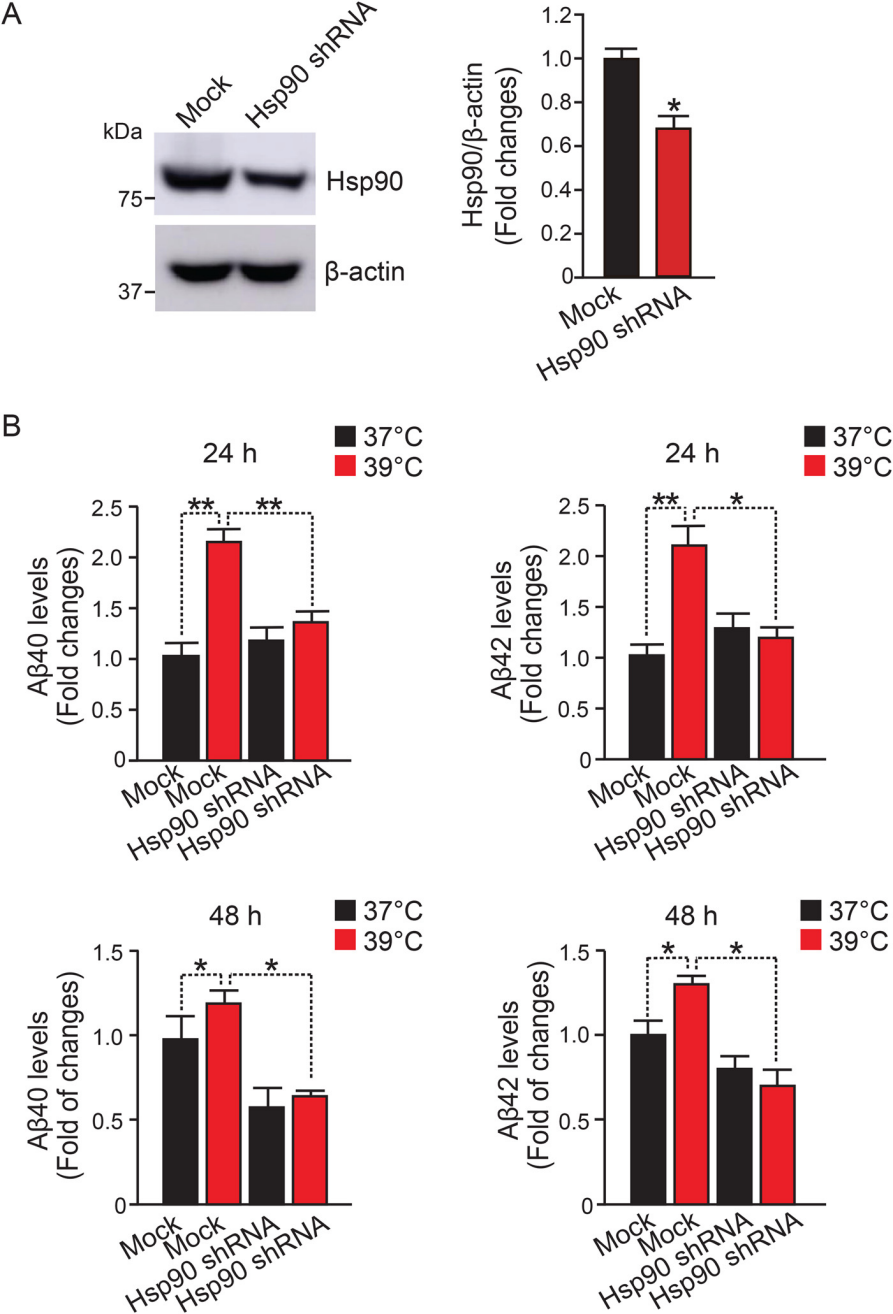
**Knockdown of Hsp90 reduces Aβ production at high temperature.***A*, HEK-APP cells were infected with lentivirus expressing Hsp90 shRNA or control shRNA. Cellular Hsp90 levels were examined with Western blotting. *B*, secreted Aβ levels from the control and Hsp90 knockdown cells were measured with an Aβ ELISA kit. Levels of Aβ40 and Aβ42 were reduced by Hsp90 knockdown in HEK-APP cells at 39 °C. Data are the mean ± S.E. (*error bars*) from three independent experiments. *, *p* < 0.05; **, *p* < 0.01 as determined with one-way ANOVA with Tukey's multiple-comparison test.

To test whether the increased level of Hsp90 is essential for enhancing Aβ production at high temperature, we knocked down the expression of Hsp90 in HEK-APP cells by infecting the cells with lentivirus producing shRNA targeting Hsp90 and measured Aβ levels in the medium with ELISA. Stable Hsp90 knockdown HEK-APP cells resulted in a significant decrease in the Hsp90 expression level (Fig. 4*A*). In Hsp90 knockdown cells cultured at 39 °C for 24 h, the secretion of Aβ40 and Aβ42 was significantly decreased by 43 and 45%, respectively, compared with that of control cells with mock infection (Fig. 4*B*). The reduction in Aβ40 and Aβ42 production was also observed in the Hsp90 knockdown cells cultured at 39 °C for 48 h (Fig. 4*B*). These results suggest that Hsp90 plays a critical role in regulating the increased γ-secretase activity.

### Knockdown of Hsp90 reduces levels of γ-secretase components and complex formation at high temperature

**Figure 5:**
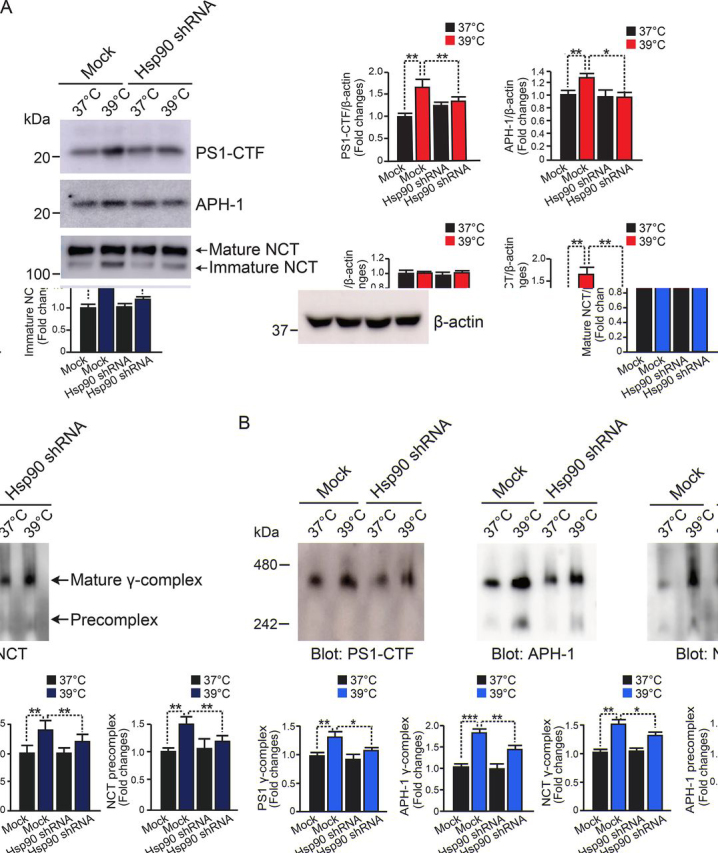
**Knockdown of Hsp90 reduces the levels of** γ**-secretase components and** γ**-secretase complex formation.***A*, the cellular levels of PS1-CTF, NCT, and APH-1 in Hsp90 knockdown HEK-APP cells at 48 h were determined with Western blotting. The levels of PS1-CTF, immature NCT, and APH-1 were significantly decreased in Hsp90 knockdown cells at 39 °C compared with control cells. *B*, 5 μg of protein from control or Hsp90 knockdown HEK-APP cells at 24 h was subjected to BN-PAGE and analyzed with Western blotting using the indicated antibodies. Formation of the mature γ-secretase complex and the APH-1/NCT precomplex was reduced in Hsp90 knockdown cells at 39 °C. Data are the mean ± S.E. (*error bars*) from three independent experiments. *, *p* < 0.05; **, *p* < 0.01; ***, *p* < 0.001 as determined with one-way ANOVA with Tukey's multiple-comparison test.

We next examined the effect of Hsp90 knockdown on levels of PS1-CTF, APH-1, and NCT in HEK-APP cells stably transfected with shRNA or mock-transfected. We found that the expression levels of PS1-CTF, immature NCT, and APH-1 were significantly decreased by 23, 28, and 18%, respectively, in Hsp90 knockdown cells compared with that of mock infection cells cultured at 39 °C for 48 h (Fig. 5*A*). With blue native PAGE (BN-PAGE), we observed lower levels of the mature γ-secretase complex (∼440 kDa) detected with the PS1-CTF antibody in Hsp90 knockdown cells compared with mock infection cells cultured at 39 °C (Fig. 5*B*, *left*). Similarly, the γ-secretase precomplex consisting of APH-1 and NCT was also decreased by knockdown of Hsp90 in the cells compared with that of mock-transfected cells cultured at 39 °C (Fig. 5*B*, *middle* and *right panels*). These results clearly demonstrated that suppression of Hsp90 in HEK-APP cells with shRNA concomitantly led to reductions in both the precomplex and mature complex of γ-secretase at high temperature (39 °C). However, this was not the case for the control temperature (37 °C). Taken together, these results suggest that Hsp90 regulates the temperature-dependent enhancement of γ-secretase complex formation and Aβ production.

### High temperature increases γ-secretase formation in vivo

**Figure 6:**
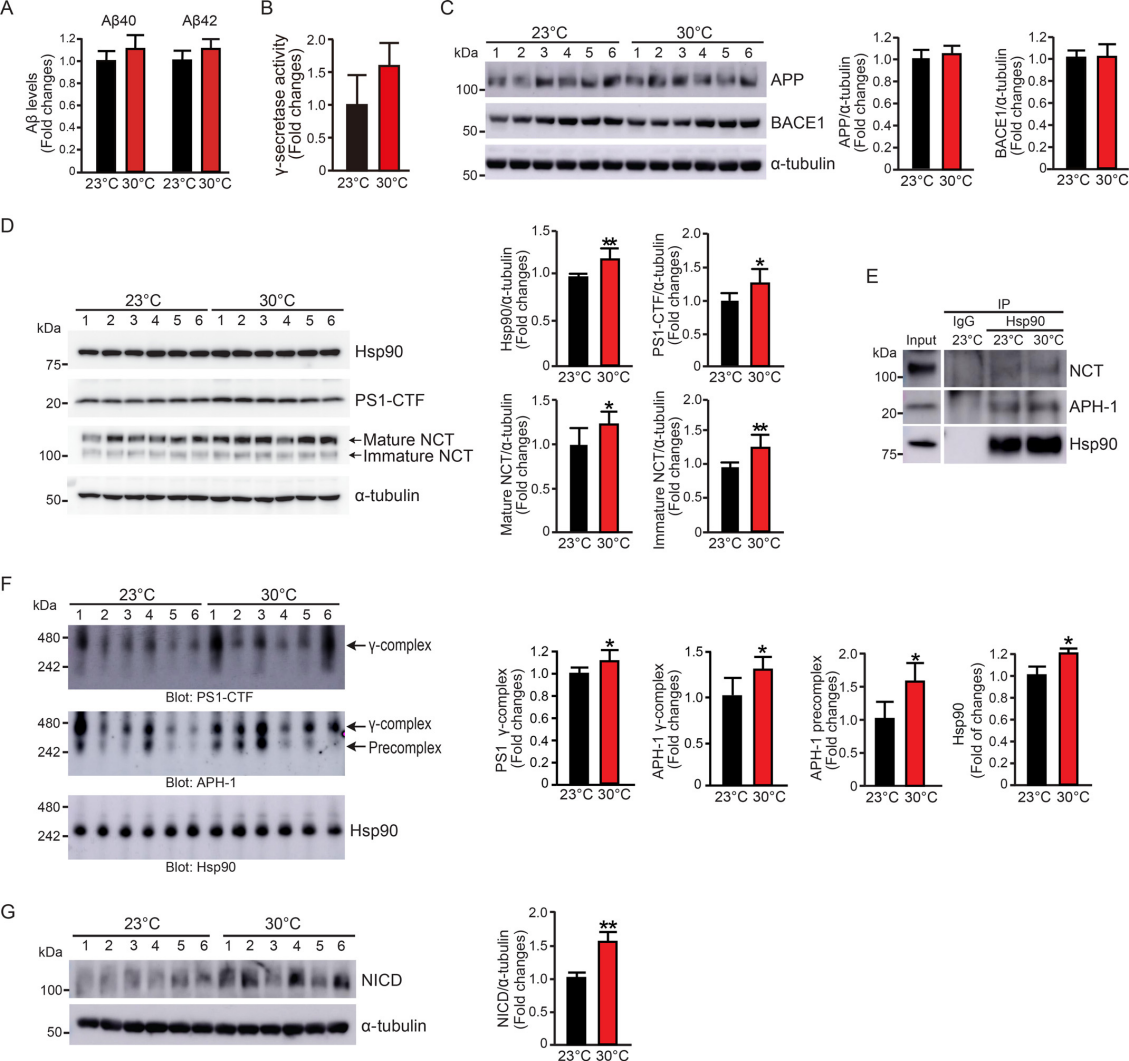
**High temperature increases** γ**-secretase complex formation and** γ**-secretase activity *in vivo*.***A*, Aβ40 and Aβ42 levels in the cortex of mice were measured with an Aβ40 or Aβ42 ELISA kit. *B*, γ-secretase activity in the cortex of mice was measured using a fluorogenic substrate. *C*, Western blotting analyses were performed with lysates from the cortical region of the brains. High temperature did not significantly alter the levels of full-length APP or BACE1. *D*, the levels of Hsp90, PS1-CTF, and NCT in the cortex of mice were detected with Western blotting. Hsp90, PS1-CTF, and mature and immature NCT were significantly increased in the cortex of mice housed at 30 °C compared with those housed at 23 °C. *E*, mouse cortex protein extracts were analyzed with IP assays using an Hsp90 antibody. High temperature increased the binding of Hsp90 to NCT and APH-1. *F*, 5 μg of protein of the cortex extracts from the mice were subjected to BN-PAGE and analyzed with Western blotting using the indicated antibodies to examine the formation of the mature γ-secretase complex, the APH-1/NCT precomplex, and the inclusion of Hsp90 in precomplex. Mice housed at 30 °C showed significantly increased formation of the premature and mature γ-secretase complex. *G*, the NICD level was significantly increased in the cortex of mice housed at 30 °C compared with those housed at 23 °C. *n* = 6 mice for each group. Data are the mean ± S.E. (*error bars*) from three independent experiments. *, *p* < 0.05; **, *p* < 0.01 as determined with Student's *t* test.

To test whether a high temperature is involved in γ-secretase formation and activity *in vivo*, we housed mice at standard room temperature (23 °C) or a higher temperature (30 °C) for 30 days. Abreu-Vieira *et al*. (21) previously reported that body temperature is significantly increased in a high ambient temperature (30–33 °C), especially in the light phase, compared with that in the standard temperature condition (22 °C) in mice. We compared Aβ40 and Aβ42 levels in the cortex of mice housed at 23 or 30 °C. Mice housed at 30 °C did not show significantly increased levels of Aβ40 or Aβ42 or γ-secretase activity in the cortex compared with those housed at 23 °C (Fig. 6, *A* and *B*). Full-length APP and BACE1 levels did not differ between the two groups (Fig. 6*C*). However, consistent with our *in vitro* studies, the levels of Hsp90, PS1-CTF, and NCT were significantly increased in the cortex of mice housed at 30 °C (Hsp90, 1.2-fold; PS-1, 1.2-fold; mature NCT, 1.3-fold; immature NCT, 1.2-fold) compared with levels at 23 °C (Fig. 6*D*). We then examined the binding of Hsp90 with NCT and APH-1 in mouse cortex extract. IP experiments revealed that the binding of Hsp90 with NCT and APH-1 slightly increased at 30 °C compared with 23 °C (Fig. 6*E*). To further confirm our findings that high temperature increased formation of the γ-secretase complex, we examined γ-secretase complex levels in the cortex of mice. We found that levels of both the precomplex and mature γ-secretase complex increased in brains of mice housed at 30 °C compared with those housed at 23 °C (Fig. 6*F*, *top* and *middle panels*). We also found that Hsp90 protein at ∼250 kDa showed a 1.2-fold increase, suggesting the inclusion of Hsp90 in γ-secretase precomplex (Fig. 6*F*, *bottom panel*).

Next, we examined the levels of Notch intracellular domain (NICD), which is generated by γ-secretase cleavage of Notch. The NICD level in the brains of mice housed at 30 °C was significantly higher (1.6-fold) than that in mice housed at 23 °C (Fig. 6*G*), suggesting that high temperature may increase the γ-cleavage of Notch. However, we cannot exclude the possibilities that high temperature could also enhance Notch ectodomain shedding or Notch substrate expression.

Taken together, our results revealed novel temperature-dependent regulation of γ-secretase formation and activity, especially *in vitro*, due to modulation of the expression levels of Hsp90 and the levels of Hsp90-bound NCT and APH-1. Although we found a small increase in γ-secretase formation in the brains of mice housed at 30 °C compared with 23 °C, this was not associated with a measurable increase in γ-secretase activity.

## Discussion

AD patients often show sleep deprivation and a raised core body temperature (22). In addition, sleep-deprived animal models of AD show a 25% increase in Aβ plaques compared with non-sleep-deprived controls (23). On the other hand, an orexin receptor antagonist increases sleep and thus decreases Aβ deposition in APP transgenic mouse models and increases the rate of Aβ clearance (24). These studies indicate that increased Aβ levels link sleep loss and AD. The disturbance of sleep and the circadian cycle leads to an increased core body temperature and increased Aβ production. Another study demonstrated age-related changes in body temperature that occurred before AD-related pathology in 3xTgAD mice (25), suggesting that elevated body temperature may be a predictor of disease or one of the earliest changes that appear after AD onset. Previous studies also showed that detergent-solubilized γ-secretase preparations incubated with a recombinant APP substrate produced increased Aβ and APP intracellular domain by increment in temperature from 35 to 45 °C, suggesting elevated γ-secretase activity (26). Another recent study showed that elevation of body temperature in APP knockin mice by lipopolysaccharide increased steady-state Aβ levels in blood plasma (27), suggesting that either the natural daily fluctuation in body temperature or the induction of fever can promote changes in Aβ generation. In this study, we found a novel regulatory mechanism of Aβ production at high temperature with participation of Hsp90 in γ-secretase complex formation. High temperature (39 °C) enhanced the interaction of Hsp90 with NCT/APH-1, thereby increasing formation of the premature and mature γ-secretase complex and then enhancing γ-secretase activity *in vitro*.

γ-Secretase cleaves C99, an intermediate APP fragment, to generate Aβ40 and Aβ42 and N-terminal APP intracellular domain. Similarly, NICD is also generated by γ-secretase cleavage (6). The four core components of the γ-secretase complex tightly regulate each other's expression, maturation, and assembly (28). The first step in complex assembly is formation of the NCT/APH-1 precomplex, which is an initial scaffold that is established prior to the generation of the full presenilin complex. The NCT/APH-1 precomplex stabilizes the γ-secretase complex. PS1-CTF binds to the NCT/APH-1 precomplex, and finally, PEN-2 completes the complex and may facilitate autocleavage of PS (29, 30). PS is the catalytic subunit of γ-secretase, and many PS mutations cause an increase in the relative amounts of Aβ42 (31). Some previous studies have reported that γ-secretase regulators such as β-arrestin, OCIaD2, Rer1p, and angiotensin receptor type 1a regulate the enzymatic activity of the γ-secretase complex (32, 33, 34, 35). In this study, we showed that Hsp90 binds to NCT and the APH-1 precomplex and then promotes formation of the mature γ-secretase complex. These results suggest that by regulating the initial steps of complex assembly, the increase in Hsp90 at high temperature controls the total level of the γ-secretase complex and, hence, its activity in cells.

Furthermore, we demonstrated that a high temperature significantly increased the levels of PS1-CTF and NCT as well as the γ-secretase complex. Interestingly, we found that only immature NCT levels were significantly increased at high temperature but not mature NCT levels in HEK-APP cells. Hsp90 knockdown in HEK-APP cells reduced the expression levels of PS1-CTF, immature NCT, and APH-1 at 39 °C to a similar level as in the 37 °C control, suggesting that PS1-CTF, immature NCT, and APH-1 levels may also be regulated by Hsp90. Thus, our studies revealed that elevation of Hsp90 at a high temperature is a key factor in the regulation of γ-secretase formation and activity during temperature stress. In our *in vivo* studies, we did not find a significant increase in endogenous Aβ40 and Aβ42 or γ-secretase secretase activity in the cortex of mice housed at 30 °C compared with those housed at 23 °C. This may be due to the low level of endogenous Aβ, which is barely detected in normal C57BL/6 mice. Alternatively, other peptide-degrading mechanisms may be activated at high temperature.

Hsp90 is a highly conserved protein with chaperone activity and plays an important role in the cell's response to stress. A previous study showed that exposure to a cold temperature (4 °C) increases the level of tau phosphorylation in 3xTg-AD mice (36). Together with our studies, these findings lead us to speculate that the stress of a temperature change, either low or high temperature, may increase the level of Hsp90 (37) and lead to increased γ-secretase complex formation and Aβ production. Hsp90 accounts for 1–2% of protein in a normal, unstressed cell; however, when cells become stressed, the level of Hsp90 increases up to 3–5% (38, 39, 40). Hsp90 is also a key player in protein processing and homeostasis by colocalizing with aggregated proteins in neurodegenerative diseases, particularly in AD and Parkinson's disease (41, 42). Among Hsp family members, Hsp90 and its cochaperone facilitate AD pathology though stabilization of an array of its client proteins (43). Previous studies showed that the binding of Hsp90 to tau promotes a conformational change and aggregation of tau protein (44, 45). Another study has shown that the levels of Hsp90 are changed in the aged human brain and contribute to the progression of aging and neurodegenerative disorders (46). Some previous studies demonstrated that heat-shock proteins may improve the pathobiology of neurodegenerative disease (47, 48). Together with our studies, these findings suggest that generation of Aβ is increased by Hsp90, but aggregation of Aβ could be suppressed by the function of other heat-shock proteins. We provide evidence showing that a temperature increase may induce stress in cells, followed by production of more Hsp90, and that excess Hsp90 is involved in development of AD. We showed that knockdown of Hsp90 expression reduces Aβ production by interfering with γ-secretase complex formation at a high temperature.

Our study provides new insight into the mechanism of regulation of the γ-secretase complex and Aβ production during temperature stress, suggesting that Hsp90 plays a role in AD pathogenesis. Furthermore, these results suggest that targeting Hsp90 and/or its interaction with APH-1 and NCT could be a key strategy for designing novel, multitargeted drugs or therapeutic strategies against AD.

## Experimental procedures

### Cell culture

HEK293T cells stably overexpressing human APP695 or C99 were cultured in Dulbecco's modified Eagle's medium at 37 or 39 °C and 5% CO_2_. All cell lines were grown in this medium supplemented with 10% fetal calf serum.

### Animal experiments

Eight-week-old female C57BL/6J mice were obtained from SLC, Inc. (Shizuoka, Japan) and maintained at the normal room temperature of 23 °C or the higher room temperature of 30 °C. Mice were provided *ad libitum* access to standard chow (CE-2, CLEA, Shizuoka, Japan) and tap water. After 4 weeks, the mice were killed by cervical dislocation, and the brains were immediately collected and frozen at −80 °C until use. The experiments in this study were performed in strict accordance with the recommendations in the Fundamental Guidelines for Proper Conduct of Animal Experiment and Related Activities in Academic Research Institutions under the jurisdiction of the Ministry of Education, Culture, Sports, Science, and Technology, Japan. The protocol was approved by the Institutional Animal Care and Use Committee at Chubu University.

### Quantification of Aβ40, Aβ42, and ApoE levels

Conditioned media were collected from HEK-APP and HEK-C99 cells at 24 and 48 h. The levels of secreted Aβ40 and Aβ42 in the conditioned media were measured with a sandwich ELISA according to the manufacturer's instructions (Wako Pure Chemical Industries, Osaka, Japan). Human Aβ and rat/mouse Aβ could be detected. The capture antibody was BNT77, and the detecting antibodies were BA27 for Aβ40 and BC05 for Aβ42. Mouse cortex for ELISA was homogenized in 10 volumes of lysis buffer, which contained 5.0 m guanidine·HCl/50 mm Tris·Cl, pH 8.0 (w/v), as described previously (49). ApoE levels in the conditioned media at 24 h were determined using an ApoE ELISA kit according to the manufacturer's instructions (MBL, Nagoya, Japan). All samples were measured in triplicate.

### Western blot analysis

HEK-APP cells or mouse cerebral cortices were washed with PBS and homogenized in lysis buffer (25 mm Tris-HCl (pH 7.6), 150 nm NaCl, 1% Nonidet P-40, 1% sodium deoxycholate, 0.1% SDS) containing a protease inhibitor mixture (Roche Applied Science). Equal amounts of protein from the cell lysate were separated with SDS-PAGE in a 5–20% gel and blotted onto polyvinylidene difluoride membranes (Sigma–Aldrich). The membranes were incubated with primary antibodies overnight at 4 °C. Appropriate peroxidase-conjugated secondary antibodies were applied, and the membranes were visualized with Super Signal Chemiluminesence (Wako). Membranes were then stripped and reprobed with anti-β-actin antibody to normalize the loading amounts. The Hsp90 antibody was purchased from BD Biosciences. The NCT, β-actin, and α-tubulin antibodies were purchased from Sigma–Aldrich. The APP (22C11) and PS1-CTF antibodies were from Millipore (Burlington, MA). The BACE1 and cleaved Notch1 antibodies were purchased from Cell Signaling Technology (Danvers, MA). The PEN-2 antibody was purchased from Abcam (Cambridge, UK). The APH-1 antibody was from Covance (Princeton, NJ).

### γ*-Secretase activity assay*

HEK-APP cells incubated at 37 or 39 °C for 48 h or mouse cortex were homogenized in 500 μl of Buffer A (150 mm KCl, 2 mm EGTA, 20 mm HEPES, pH 7.5, and phosphatase and protease inhibitors) using a homogenizer pestle. The homogenates were centrifuged at 45,000 rpm at 4 °C for 1 h. The pellets were washed and homogenized again with 500 μl of Buffer A on ice and then centrifuged at 45,000 rpm at 4 °C for 1 h. The pellets were homogenized with 270 μl of Buffer A containing 1% CHAPSO and then rotated at 4 °C for 2 h to solubilize membrane. The samples were centrifuged at 45,000 rpm for 1 h at 4 °C, and the supernatant was collected. 100 μg of protein from HEK-APP cells and 50 μg of protein from mouse cortex were used for γ-secretase assay. 8 μm γ-secretase fluorogenic substrate (Sigma–Aldrich) was added into samples with or without 20 μm DAPT, and the samples were incubated in γ-secretase assay buffer (100 mm Tris-HCl, pH 6.8, 4 mm EDTA, 0.5% CHAPSO) at 37 or 39 °C for 1–2 h (50). The values were measured by a plate reader (SPECTRA MAX GEMINI EM, Tokyo, Japan).

### Cell surface biotinylation

Cell surface biotinylation was carried out using a cell surface protein isolation kit (Pierce) (51). Briefly, HEK-APP cells cultured at 37 or 39 °C for 24 h were washed twice with PBS and incubated in 10 ml of 0.25 mg/ml Sulfo-NHS-SS-Biotin in PBS for 30 min at 4 °C. The cells were scraped and washed twice with TBS (10 mm Tris/HCl (pH 7.5) and 150 mm NaCl) and lysed in the lysis buffer containing protease inhibitors. Each lysate was incubated with streptavidin-agarose beads at 4 °C for 30 min, and captured proteins were eluted with 10 mm DTT.

### BN-PAGE

HEK-APP cells or mouse cortices were homogenized in a native sample buffer (Thermo Fisher Scientific) containing 1% digitonin and a protease inhibitor mixture. After centrifugation at 20,000 × *g* at 4 °C for 30 min, the supernatant was separated on a 4–16% BisTris gel (Thermo Fisher Scientific) according to the instructions of the Novex BisTris gel system (Thermo Fisher Scientific). The γ-secretase complex levels were detected with PS1-CTF, APH-1, and NCT antibodies.

### IP assay

HEK-APP cells or mouse cortex were lysed with lysis buffer containing 1% digitonin followed by centrifugation at 20,000 × *g* for 30 min at 4 °C. All IP steps were performed at 4 °C. Cell lysates were immunoprecipitated overnight with anti-Hsp90 IgG or control IgG (Santa Cruz Biotechnology, Inc., Dallas, TX) in the presence of protein G-Sepharose (Thermo Fisher Scientific). The beads were washed five times with lysis buffer. The samples were subjected to 5–20% gradient SDS-PAGE and transferred to a polyvinylidene difluoride membrane for Western blotting analysis.

### Immunostaining

After HEK-APP cells were incubated at 37 or 39 °C for 48 h, they were fixed in 4% paraformaldehyde. Next, they were permeabilized with 0.1% Triton X-100 and blocked for 45 min in 10% normal goat serum in PBS. Fixed cells were incubated with primary antibody (Hsp90 and APH-1 or NCT) for 12 h at 4 °C. Immunofluorescent labeling was carried out with Alexa Fluor 488– and Alexa Fluor 568–tagged secondary antibodies (Invitrogen). To assess cell surface APP internalization, immunostaining was performed as reported previously (51). Images were captured on a confocal microscope (Olympus FV 3000) (Olympus, Tokyo, Japan) using an oil-immersion plan Apo ×60 A/1.40 numerical aperture objective lens. ImageJ (National Institutes of Health) was used for quantification of the colocalization Hsp90 with NCT or APH-1. Threshold intensity was preset for both fluorescent signals, which was determined with the thresholding function. The colocalized pixels above the threshold intensity were automatically quantified and scored, which was expressed as colocalized mean intensity positive for both channels. The colocalization was presented as the percentage of the colocalized intensity relative to total fluorescence intensity.

### Hsp90 knockdown

HEK-APP cells were cultured in 6-well plates at a density of 1 × 10^5^ cells/well and allowed to adhere for 24 h before infection. Cells were infected with lentivirus containing shRNA directed against human Hsp90 (Hsp90-shRNA) (Santa Cruz Biotechnology, Inc.) or nontargeting vector-control shRNA (NC-shRNA) (Santa Cruz Biotechnology) in the presence of 5 μg/ml Polybrene. At 48 h post-transfection, cells were selected with 5 μg/ml puromycin for 10–15 days to obtain Hsp90 knockdown cells.

### Statistical analysis

Statistical analysis was performed using a statistical package, GraphPad prism 7.0 software (GraphPad Software, San Diego, CA). All values are presented as the mean ± S.E. of at least three independent experiments. Student's *t* test was used to determine whether the results were significantly different between two groups. We compared group difference with one-way analysis of variance (ANOVA) followed by Tukey's multiple-comparison test for two or more groups against a control group. A *p* value of <0.05 was considered to represent a significant difference.

## Data availability

All data are included in the article.
